# Access to prompt diagnosis: The missing link in preventing mental health disorders associated with neglected tropical diseases

**DOI:** 10.1371/journal.pntd.0007679

**Published:** 2019-10-17

**Authors:** Albert Picado, Sarah Nogaro, Israel Cruz, Sylvain Biéler, Laura Ruckstuhl, Jon Bastow, Joseph Mathu Ndung’u

**Affiliations:** 1 Foundation for Innovative New Diagnostics (FIND), Geneva, Switzerland; 2 National School of Public Health, Instituto de Salud Carlos III, Madrid, Spain; Universiteit Antwerpen, BELGIUM

## Introduction

Globally, there are an estimated 1 billion people suffering from at least one of the 20 neglected tropical diseases (NTDs) prioritized by the World Health Organization (WHO). Prevalent in tropical and subtropical regions, this group of NTDs comprises diverse diseases, including vector-borne parasitic diseases (such as human African trypanosomiasis [HAT], Chagas disease, and leishmaniasis), skin diseases caused by environmental bacteria (such as Buruli ulcer [BU]), foodborne parasitic diseases (such as taeniasis/cysticercosis) or snake bite envenoming, which—together with scabies and other ectoparasites, mycetoma, and deep mycoses—were recently added to the list [1]. Despite their differences, NTDs are synonymous with poverty, life-long disability, stigma, and discrimination, not to mention the lack of effective control tools such as vaccines, diagnostics, and drugs.

The majority of NTDs have been shown to worsen a patient’s mental health [2,3] in different ways: (i) neurological—infection directly affects the brain, causing mental disorders, such as in HAT, Chagas disease, and neurocysticercosis [4]; (ii) pain or physical impairment—large skin ulcers in BU, blindness in onchocerciasis, and scrotal swelling in lymphatic filariasis, all of which reduce the quality of life and increase the risk of depression [5]; and (iii) stigma and social discrimination—NTDs are stigmatizing due to their symptoms, e.g., skin lesions and disfigurement in BU, cutaneous leishmaniasis (CL), or leprosy, as well as their association with poverty. All these factors are related to impaired quality of life and have psychological consequences [6].

New initiatives, such as the Mental Wellbeing and Stigma (MWS) Task Group from the Neglected Tropical Disease Non-Governmental Organization Network, are working to raise awareness of psychosocial morbidity in patients and communities affected by NTDs [7]. The ultimate objective of the MWS group is to improve the care of both NTD patients and their caregivers who are suffering or are at risk of suffering from mental health issues. This line of action is crucial but would be considerably more effective if complemented by preventive measures. Preventing NTDs in the first place will reduce the risk of suffering from the mental health consequences associated with them. Alone or in combination, the following interventions should be encouraged to control NTDs, mitigate their impact, and promote well-being for affected communities [8]: (i) preventive chemotherapy through mass drug administration campaigns (for soil-transmitted helminthiasis and schistosomiasis); (ii) vector control (for Chagas disease, HAT, dengue, and chikungunya virus); or (iii) providing safe water, sanitation, and hygiene (WASH) to control trachoma, soil-transmitted helminths, and lymphatic filariasis.

In this Viewpoint, we suggest a complementary strategy to reduce the potential onset of mental health disorders among NTD patients by advocating for early diagnosis and therefore prompt treatment of these diseases. In NTDs, the development of neuropsychiatric manifestations is usually associated with chronicity and late-stage disease. The parasites causing HAT or Chagas disease only invade the central nervous system (CNS) in the so-called second and chronic stages of the disease. Skin NTDs, such as BU, CL, or yaws, often start as painless swelling or small nodules that eventually develop into disfiguring and disabling ulcers if they are not treated early. Similarly, long-term infections are associated with disability and chronic pain as experienced by patients suffering from leprosy, lymphatic filariasis, and mycetoma or blindness in the case of nontreated eye infections with bacteria (trachoma) and parasites (onchocerciasis). The latter has also been associated with nodding syndrome, a neurological condition causing progressive neurological deterioration and epilepsy in children. We argue that treating NTDs as soon as possible after infection will reduce the risk of developing neuropsychiatric disorders. Treatment in the early stage of disease requires prompt access to correct diagnosis. In this paper, we present three examples showing that developing and implementing better tools and strategies to diagnose NTDs could significantly reduce their adverse impact on mental health.

## HAT

HAT, or sleeping sickness, is a parasitic disease caused by protozoans belonging to the species *Trypanosoma brucei*. The large majority of cases are caused by *T*. *b*. *gambiense*. The disease has 2 consecutive stages: the first a hemolymphatic stage and the second a meningo-encephalitic stage. In the first stage, trypanosomes invade the hemolymphatic system, causing clinical signs and symptoms that are not specific to the disease (e.g., headaches or fever). If the patient is not treated, the trypanosomes invade the CNS (stage 2), where they cause damage resulting in neuropsychiatric disorders (e.g., sleep disorders, derangement, or deep sensory disturbances). These syndromes are associated with a strong stigma, which affects patients and impacts their health-seeking behavior [9]. Diagnosing and treating HAT patients in stage 1 precludes the development of mental health problems associated with stage 2. However, diagnosing HAT can be challenging because the clinical signs and symptoms in stage 1 are not specific to the disease.

Until recently, diagnosis was conducted by specialized teams, often as part of large active screening campaigns (teams of 10 to 15 people) or in a limited number of health facilities, because case confirmation (usually compulsory to start treatment) requires trained laboratory personnel. The development of rapid diagnostic tests (RDTs) to screen for *T*. *b*. *gambiense* HAT [10,11] has enabled implementation of new control strategies. First, RDTs can be deployed in primary healthcare facilities, enhancing the capacity for passive screening for HAT [12]. Reducing the distance that a sick person has to travel to get screened for HAT reduces the time from onset of symptoms to treatment and allows diagnosing a significant number of HAT cases in the first stage of the disease. In Kongo Central province (Democratic Republic of the Congo), for example, 65.4% of the HAT cases diagnosed in health facilities using RDTs were in the early stage of the disease [13]. Second, RDTs allow implementing targeted active screening using smaller teams (e.g., 1 to 2 people using motorcycles [14] to easily cover distances). These new active screening strategies (door-to-door) are more efficient than active mass screening in low-prevalence settings [15] and are currently being implemented using RDTs in Guinea, Côte d’Ivoire, and Chad. Clearly, diagnosing HAT patients earlier improves the outcome of their treatment and contributes to the control and elimination of the disease in endemic countries.

## CL

CL is one of the clinical forms of leishmaniasis, a group of diseases caused by a protozoan of the *Leishmania* genus. Although CL is not lethal, it causes chronic and disfiguring skin lesions, leading to well-documented psychosocial morbidity in affected individuals [2,3]. The impact of CL on the mental health of the patients depends largely on the type and location of the lesions [16], particularly for women and young girls if the lesions occur on the face [17]. Early treatment prevents the enlargement of the scars and disfigurement, as well as the risk of developing mucocutaneous leishmaniasis, a secondary form of CL that causes significant tissue destruction. This form is endemic in some areas, such as South America. Because treatment for CL is toxic and painful, it should only be initiated after a confirmatory diagnosis has been made. However, CL diagnosis is still based on demonstrating the presence of *Leishmania* parasites by direct microscopy of Giemsa-stained smears made using skin scrapings or fine needle aspirates obtained from lesions. This approach reduces access to diagnosis and treatment at reference health facilities, such as district hospitals in endemic countries. In Afghanistan, for example, where almost 30,000 CL cases were reported in 2015, CL patients from rural areas had to travel to the reference leishmaniasis clinic in Kabul for diagnosis and treatment. The lack of diagnostic capacity in peripheral health facilities severely delays or precludes access to care for patients, leading to chronic skin lesions, reduced function, disability, stigma, and mental health problems.

An RDT for the detection of *Leishmania* in CL skin lesions is now available: the CL Detect Rapid Test (InBios International, Seattle, WA). This RDT allows diagnosing CL in primary healthcare facilities as it only requires obtaining a sample from the lesion using a small dental broach; the results are available in less than an hour and can be interpreted the same way as results from a malaria RDT. As shown in different studies, the specificity of the CL Detect RDT is good, but its sensitivity is low [18–20]. Nevertheless, the CL Detect can significantly improve access to CL diagnosis in endemic regions. Based on its performance, people showing signs of having CL and who then test positive using the RDT in peripheral health facilities should start treatment without delay. Those who test negative still need to be referred for confirmation of either being positive or negative [19]. Better diagnostic tests for CL should be developed [21], but we must maximize the use of the tools already available, in particular, the CL Detect RDT, to improve access to CL care and reduce the mental health sequelae associated with CL in endemic countries such as Afghanistan.

## BU

BU is an important public health problem among rural communities in several countries in sub-Saharan Africa, where it mainly affects children under 15 years of age. As with several other NTDs, BU initially presents as small, nonspecific, painless swellings that, without treatment, eventually ulcerate and enlarge into disfiguring sores that cause long-term functional disability in up to 25% of cases. With the majority of cases impacting children, patients typically have to be accompanied by family members during several weeks of hospitalization, which has a negative effect on household earnings and leads to many patients being abandoned in hospitals or withdrawn from treatment. Patients with large ulcers or cured people with disfigurement often become socially excluded through negative attitudes in the community and end up dropping out of school or from the workforce [22]. Early and accurate diagnosis and antibiotic treatment are highly effective and therefore minimize the suffering, disability, and socioeconomic burden of the disease. Thus, it is crucial to have a radically improved diagnostic test that can make decentralized early diagnosis possible [23].

Although current diagnostics for BU are expensive and inappropriate for poor, rural settings, significant progress has been made in recent years. Simple molecular amplification methods, such as the loop-mediated isothermal amplification (LAMP), are now available for BU diagnosis [24]. However, even if these tests are easier to perform than PCR, they still require basic laboratory infrastructure. To allow BU diagnosis at primary healthcare facilities in endemic regions, efforts have been concentrated in developing an RDT to detect mycolactone, an exotoxin produced by *Mycobacterium ulcerans* in BU lesions [23]. Promising preliminary results of a prototype RDT to detect mycolactone were presented at the WHO meeting on “BU and skin NTDs” held in Geneva from 25–27 March 2019. This point-of-care test will enable the identification of the disease in its early stages at community or primary healthcare facilities where the at-risk populations live. As for CL, early diagnosis and treatment of BU patients will limit the extent of the skin lesions, disability, and stigma, thus significantly reducing the adverse impact on mental health.

## Conclusion

**Fig 1:**
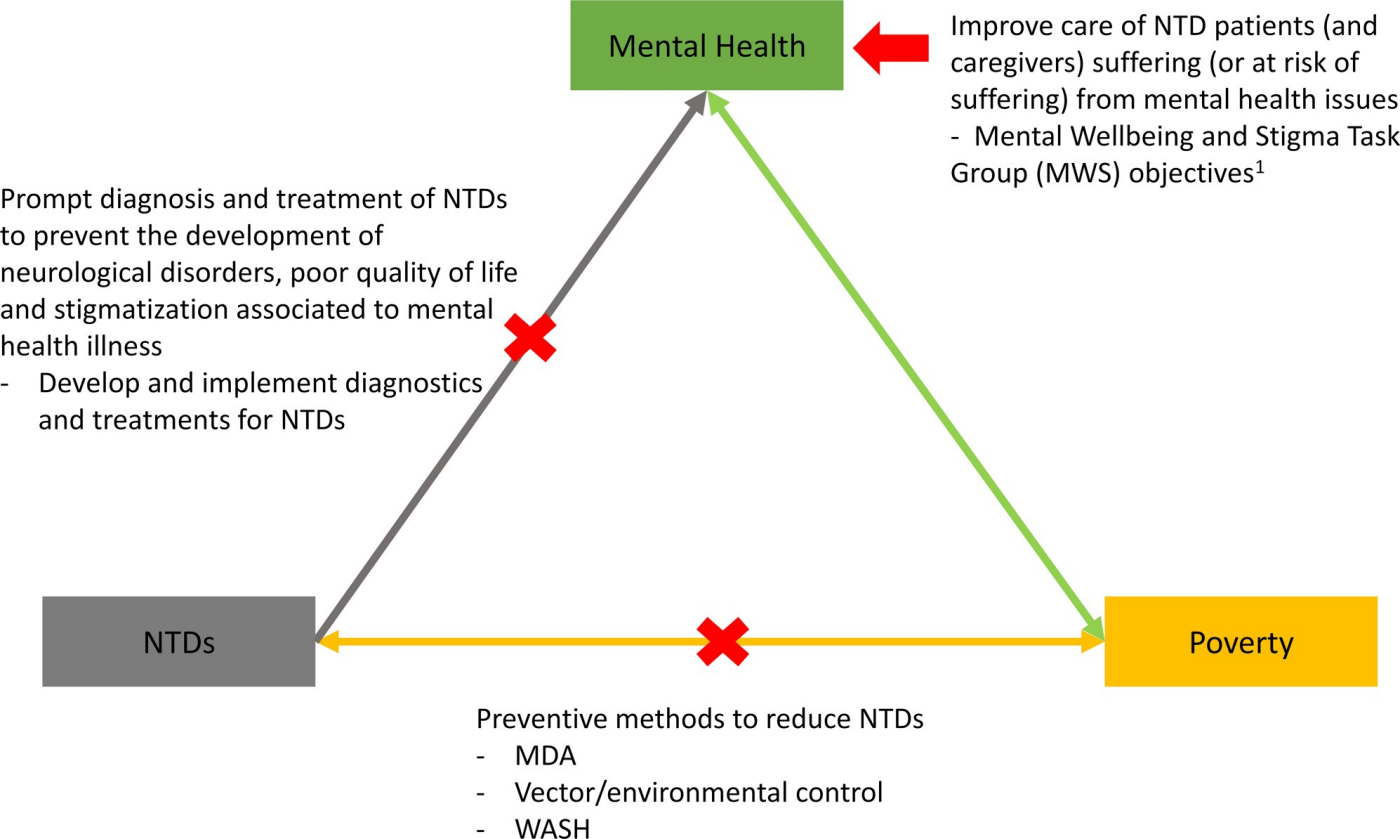
Poverty, NTDs, and mental health cycle, plus areas of intervention. ^1^MWS group objectives: “(i) to support and develop resources for advocacy of affected individuals and to promote their empowerment; (ii) to ensure NTD programmes include interventions which promote positive attitudes and behaviour of communities to those affected and address structural discrimination; (iii) to promote self-advocacy and expression of the needs of those affected; and (iv) to increase the awareness of the rights and responsibilities of those affected and those who provide services to them, including their caregivers” [3]. MDA, mass drug administration; MWS, Mental Wellbeing and Stigma; NTD, neglected tropical disease; WASH, water, sanitation, and hygiene.

The links between poverty, mental health, and NTDs are well established (Fig 1). Preventing NTDs, ensuring prompt access to diagnosis and treatment of NTDs, and improving care of those suffering from the psychological consequences of these diseases should be part of a holistic approach to break the existing vicious cycle of disease—lack of diagnosis, lack of treatment, and deterioration. As illustrated by the previous examples, the efforts to improve diagnosis of NTDs should be made at different levels: research and development (e.g., RDT for BU), evaluation (e.g., RDT for CL), and implementation (e.g., RDT for HAT). The burden of neuropsychiatric disorders, particularly for NTDs, is sadly underestimated [2]. Thus, even if the impact of prompt diagnosis of NTDs in preventing mental health disorders remains to be evaluated, we can foresee that it is significant.
